# Developing tailored theoretically informed goal-setting interventions for rehabilitation services: a co-design approach

**DOI:** 10.1186/s12913-022-08047-6

**Published:** 2022-06-22

**Authors:** Amanda Baker, Petrea Cornwell, Louise Gustafsson, Claire Stewart, Natasha A. Lannin

**Affiliations:** 1 School of Allied Health Sciences, Griffith University, Brisbane, Australia; 2 Clinical Excellence Division, Statewide Rehabilitation Clinical Network, Queensland Health, Brisbane, Australia; 3 Physiotherapy Department, Sunshine Coast University Hospital, Allied Health, Sunshine Coast Hospital and Health Service, Sunshine Coast, QLD Australia; 4 Department of Neuroscience, Central Clinical School, Monash University, Melbourne, Australia; 5 Alfred Health, Melbourne, Australia

**Keywords:** Behaviour change, Co-production, Decision-making Shared, Goals, Implementation Science, Intervention design, Occupational Therapy, Physical Therapists, Stroke Rehabilitation, User centred design

## Abstract

**Background:**

Several active ingredients contribute to the purposes and mechanisms of goal-setting in rehabilitation. Active ingredients in the goal-setting process include, interdisciplinary teamworking, shared decision-making, having meaningful and specific goals, and including action planning, coping planning, feedback, and review. Clinicians have expressed barriers and enablers to implementing these active ingredients in rehabilitation teams. Interventions designed to improve goal-setting practices need to be tailored to address context specific barriers and enablers. Attempts to understand and enhance goal-setting practices in rehabilitation settings should be supported using theory, process models and determinant frameworks. Few studies have been undertaken to enhance goal-setting practices in varied case-mix rehabilitation settings.

**Methods:**

This study is part of a larger program of research guided by the Knowledge to Action (KTA) framework. A multisite, participatory, codesign approach was used in five sites to address three stages of the KTA. (1) Focus groups were conducted to understand barriers and enablers to implementing goal-setting at each site. Following the focus groups three staff co-design workshops and one consumer workshop were run at each site to (2) adapt knowledge to local context, and to (3) select and tailor interventions to improve goal-setting practices. Focus groups were analysed using the Theoretical Domains Framework (TDF) and informed the selection of behaviour change techniques incorporated into the implementation plan.

**Results:**

Barriers and enablers identified in this study were consistent with previous research. Clinicians lacked knowledge and understanding of the differences between a goal and an action plan often confusing both terms. Clinicians were unable to demonstrate an understanding of the importance of comprehensive action planning and review processes that extended beyond initial goal-setting. Interventions developed across the sites included staff training modules, a client held workbook, educational rehabilitation service flyers, interdisciplinary goal-based case conference templates, communication goal boards and a key worker model. Implementation plans were specifically established for each site.

**Conclusions:**

Rehabilitation teams continue to struggle to incorporate a truly client-centred, interdisciplinary model of goal-setting in rehabilitation. Whilst clinicians continue to lack understanding of how they can use aspects of goal-setting to enhance client outcomes and autonomy in rehabilitation settings.

**Supplementary Information:**

The online version contains supplementary material available at 10.1186/s12913-022-08047-6.

## Contributions to the literature’ section.

- This study adds an Australian context to the current literature examining barriers and enablers to implementing goal-setting practice in rehabilitation services. Furthermore this study describes the challenges faced by clinicians working in varied case mix rehabilitation services.
- This study describes a comprehensive approach to goal-setting intervention development and implementation that can be replicated in clinical practice.
- This research is based on a theoretical conceptual framework and has led to development of six interventions aiming to comprehensively address the active ingredients influencing goal-setting practices in rehabilitation services.

## Background

The Australian Faculty of Rehabilitation Medicine (AFRM) recommend that "*the patient and the rehabilitation team work together to establish meaningful and achievable treatment goals*” in rehabilitation services [1] (p.6). Goal-setting has been defined as the establishment or negotiation of rehabilitation goals [2] and is recognized as a complex process that has the potential to enhance client outcomes and autonomy [3]. The Medical Research Council (MRC) highlight that to understand how a complex intervention works, one must first identify the ‘active ingredients’ [4]. Whilst purposes and mechanisms for goal-setting in rehabilitation are varied [5], active ingredients in the goal-setting process include, interdisciplinary teamworking [6, 7], shared decision-making (SDM) [8], setting specific and meaningful goals [9] and incorporating action planning, coping planning, feedback and review [10, 11]. Furthermore, these active ingredients are required to be implemented collaboratively by large teams of diverse professionals, clients, and families [5, 12]. Evidence to date demonstrates that not all active ingredients in rehabilitation goal-setting are consistently implemented in clinical practice [11, 13–15]. A previous study in this program of research found that rehabilitation clinicians set poorly defined goals as individual disciplines, and included limited action planning and review of goal progress [11].

The evidence practice pipeline by Glasziou and colleagues [16] highlights reasons why evidence is not implemented into clinical practice. Firstly, implementation can only occur if clinicians are aware of and accept the evidence. In rehabilitation, clinicians have previously demonstrated a lack of knowledge about the evidence for goal-setting in rehabilitation [17]. Secondly, clinicians must accept the evidence is relevant to their practice and have the capability to implement the evidence [16]. In rehabilitation, clinicians have demonstrated differing beliefs about the importance and timing of goal-setting and demonstrated a lack of skill in implementing aspects of goal-setting such as shared decision-making [8, 18–20]. The final step in the evidence practice pipeline [16] is for clinicians to act on and implement the evidence with clients who have the capability of adhering to the recommendations [16]. Factors affecting clinicians’ ability to implement goal-setting in rehabilitation settings include nursing shift work [8, 21], staff turnover [21] and competing priorities such as discharge planning [21]. Furthermore, changes to a client’s cognitive and communication abilities influence clinicians’ beliefs about the capability of rehabilitation clients to participate in goal-setting [8, 20, 21]. Research into barriers and enablers to implementing goal-setting in rehabilitation has predominantly been conducted with condition specific population groups in the UK, Europe and New Zealand limiting the generalisability of findings to varied case-mix rehabilitation services in Queensland. It has been proposed that tailoring interventions and addressing context specific barriers and enablers improves the likelihood of successful implementation of evidence into practice [4].

Implementing evidence into clinical practice requires careful planning using structured process models and determinant frameworks to support clinicians to change their practice [22]. The Knowledge to Action (KTA) framework is one process model that is simple to operationalise and aligns with existing quality improvement cycles within healthcare [23]. Phases of the KTA can occur iteratively and include, synthesising the evidence base, determining the evidence-practice gap, identifying barriers and enablers, adapting knowledge to context, selecting and tailoring interventions, monitoring intervention implementation and evaluating and sustaining the change. At the centre of the KTA framework, lies the development and synthesis of knowledge. Several systematic reviews on goal-setting in rehabilitation have already been published [2, 8, 24–26]. Moving to the seconds stage of the KTA framework the first study in this program of research used medical record audits and client interviews to highlight evidence practice gaps in goal-setting practices across varied case mix rehabilitation services in Queensland [11]. Evidence-practice gaps included limited shared decision-making, a focus on therapist-led goal-setting within a multidisciplinary model, limited inclusion of meaningful activity and participation goals, limited specificity of goal statements and limited action planning, feedback and review [11].

The subsequent three stages of the KTA framework: identifying barriers and enablers, adapting knowledge to context, selecting and tailoring interventions, are the focus of this study. There are several determinant frameworks that support analysis of barriers and enablers including the Theoretical Domains Framework (TDF) [27]. The TDF was originally designed to understand health professional’s behaviour and promote understanding of barriers and enablers to implementing evidence [27]. The TDF is often used in conjunction with the Behaviour Change Wheel (BCW) framework that identifies the intervention functions (intervention functions include a broad range of categories by which an intervention can change behaviour) and behaviour change techniques (BCT) (an active component of an intervention designed to change behaviour) [28] that are likely to overcome specific barriers to implementation. How an intervention is developed may impact the uptake and implementation of an intervention in clinical practice. Incorporating both clients and clinicians into the co-design of research may improve intervention uptake through enhanced awareness, acceptance of the evidence and ownership of the intervention [29]. Therefore, unlike previous goal-setting studies, a participatory co-design approach was used to develop the interventions in this study.

In addition to how an intervention is developed it is important to consider how an intervention will be implemented ensuring it is clear exactly what behaviour needs to change i.e. who, will do what, when, where and how [30]. To address known barriers, and to implement a clearly defined behaviour, behaviour change techniques such as facilitation, prompts, audit and feedback promote problem solving and enhance uptake of interventions in the clinical setting [31, 32]. Facilitation roles involve individuals with the appropriate skills and knowledge to support clinicians through change, these roles can be both external (i.e. external researchers) or internal (i.e. local team leaders) [32]. Audit and feedback may be more effective when delivered by a colleague or supervisor and when action plans are developed to act on the feedback provided [31]. Incorporating facilitation roles, audit and feedback within implementation plans can ensure interventions are implemented as intended, that implementation is closely monitored and that clinicians are supported to agree and act on the evidence with clients [33].

This study uses a theoretically informed conceptual framework [11], following an implementation process model (KTA) and determinant framework (TDF) to progress previous research [11] and support the understanding of barriers and enablers in varied case-mix rehabilitation services. Therefore, the aim of this study is to analyse site specific barriers and enablers to implementing goal-setting and to co-design tailored interventions and implementation plans to address the evidence practice gap surrounding inclusion of goal-setting ‘active ingredients’ across five varied case-mix rehabilitation services in Queensland, Australia. This study will clearly describe the detail of intervention development to allow clinicians to replicate this research in clinical practice [34].

## Methods

### Study design

**Fig. 1:**
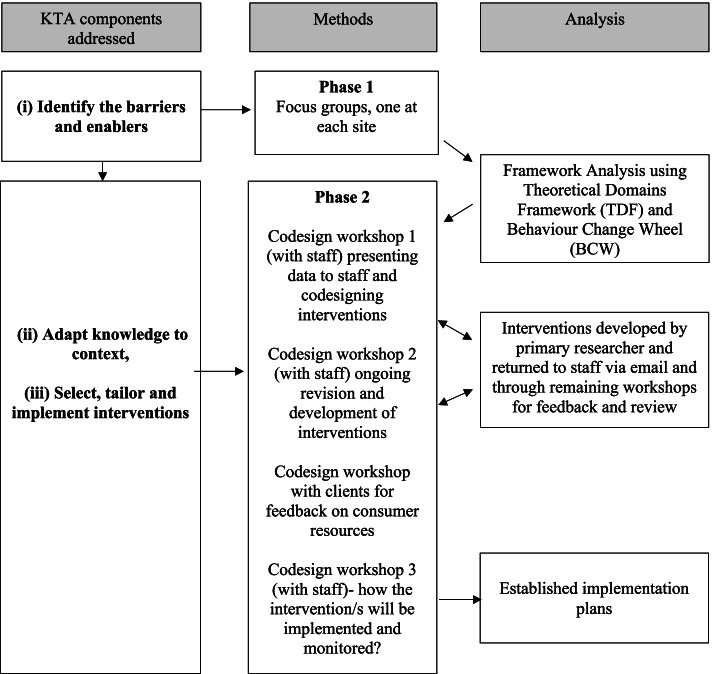
Process for developing theoretically informed, tailored site-specific goal-setting interventions

This participatory co-design, multisite study is part of a larger program of research guided by the Knowledge to Action Framework (KTA). Two distinct research phases were designed to address three stages of the KTA framework: (i) identifying barriers and enablers, (ii) adapting knowledge to the context, and (iii) selecting and tailoring interventions and implementation plans to improve client-centred goal-setting practices (Fig. 1). In this paper, the methods and results of phase 1 are presented prior to the methods and results of phase 2, consistent with the sequential nature of the research.

### Study sites

Three inpatient and two community rehabilitation services volunteered to participate in this study via an emailed expression of interest. These services are representative of local Queensland public rehabilitation services with diverse multidisciplinary teams. Details of service demographics have been presented elsewhere [11]. Institutional ethical clearances were obtained from The Prince Charles Hospital and Health Service Human Research Ethics Committee and Griffith University Ethics Committee prior to commencing. The better reporting of interventions: template for intervention description and replication checklist (TIDieR) was used to guide reporting of this study (Supplementary Material).

### Phase 1- identify barriers and enablers—methods and results

#### Participants

Clinicians, including people in leadership roles, from medical, nursing and allied health were eligible to participate if they were involved in goal-setting practices within their service. No other exclusion criteria were applied. A maximum number of 13 clinicians were purposively selected to participate in one focus group at each site. Participant numbers were selected based on feedback from each of the rehabilitation teams, there was no minimum number of participants identified. All clinicians were required to provide informed signed consent.

#### Procedure

Focus groups were selected as the method of choice to best understand clinician’s experiences of implementing goal-setting and to specifically uncover why teams have faced challenges in the past. Focus groups also enabled the exploration of interactions between team members and offer the opportunity to pursue further questioning strategies as new themes arise [35]. Focus groups were predominantly facilitated by the primary researcher, as the primary researcher is a novice qualitative researcher, a second experienced researcher was present to assist in facilitating the discussions and collecting field notes. Each focus group was conducted in person and a topic guide was developed to support the focus groups (Supplementary material). The questions investigated the clinician’s perspectives of the meaningfulness of goal-setting, the current goal-setting practice and the perceived barriers and enablers to implementing goal-setting in each service. Focus groups lasted for between 40–60 min, were audio recorded, deidentified and transcribed verbatim for analysis.

#### Data analysis

All focus group transcripts were imported into Nvivo 12 software. Four focus groups were analysed by two coders (AB and CS) and the final focus group by the primary researcher (AB). Focus group transcripts were deductively coded to two different frameworks. Firstly, focus groups were mapped to the active goal-setting ingredients (interdisciplinary teamworking, shared decision-making (SDM), specific and meaningful goals and incorporating action planning, coping planning, feedback and review [11]) and secondly mapped to the barriers and enablers according to the TDF. Discrepancies were discussed with an additional member of the research team (NL) and themes were agreed upon.

#### Results

Focus group clinicians represented a wide range of disciplines, participant demographics are presented in Table 1. Barriers and enablers to implementing the active ingredients of goal-setting practices are summarised below with reference to domains of the TDF (Table 2).

**Table 1:** Focus group participants

Site	Disciplines represented	Sex	Average Age	Average years of experience in the profession	Average years of experience in rehabilitation
Site 1 (*n* = 7) (*n* = 1 missing data point)	Dietetics, Nursing, Occupational Therapy, Physiotherapy, Rehabilitation Consultant, Speech Pathology	100% female	34.17 (± SD 6.40)	11.17 (± SD 6.55)	7.17 (± SD 4.31)
Site 2 (*n* = 9) (*n* = 1 missing data point)	Neuropsychology, Nursing, Occupational Therapy, Physiotherapy, Rehabilitation Consultant, Social Work, Speech Pathology	100% female	41.38 (± SD 9.52)	16.88 (± SD 9.40)	12.63 (± SD 8.55)
Site 3 (*n* = 7)	Neuropsychology, Nursing, Occupational Therapy, Physiotherapy, Rehabilitation Consultant, Speech Pathology	29% male	37.43 (± SD 9.88)	13.71 (± SD 9.34)	9.86 (± SD 6.23)
		71% female			
Site 4 (*n* = 13) (*n* = 2 missing data points)	Allied Health Assistant, Dietetics, Neuropsychology, Nursing, Occupational Therapy, Physiotherapy, Speech Pathology	8% male	38.82 (± SD 10.41)	12.18 (± SD 10.82)	7.82 (± SD 5.74)
		92% female			
Site 5 (*n* = 8)	Dietetics, Occupational Therapy, Physiotherapy, Social Work, Speech Pathology	12.5% male	41.75(± SD 9.62)	17 (± SD 10.44)	12.52 (± SD 10.44)
		87.5%female

**Table 2:** Summary of Focus group key findings- Barriers and enablers to goal-setting in Queensland Rehabilitation Services

Active ingredients	TDF domain	Key themes	Quotes
Teamworking	Knowledge	Lack of understanding of a client centred goal-setting focus (.^b^)	*“How do we separate out the individual discipline goals? For example, you know, be able to pronounce this, or be able to match words to pictures with 50 per cent accuracy … How do we distinguish from that from the shared goals?” (Site 3)*
	Skills	Skills to set rehabilitation goals with clients outside of discipline skill sets (.^b^)	*“I could set a broad goal, but it probably wouldn’t be… I wouldn’t have enough insight to have a SMART goal for that.”* (Site 3)
	Social and professional role and identity	Absence of the nursing role (inpatient rehabilitation) (.^b^)	*“we don’t get included…in the MDT, or the allied health goal-setting,”* (Site 1)
	Environmental Context	The need for consistent processes (^a^) but the want for flexibility (.^b^)	*“I think the challenge is always trying to find one process, and one tool that everyone is going to use… but if we really want to be person-centred, then we need to be flexible about how we do our goal-setting”* (Site 4)
		The case conference forum (.^a^)	*“case conference is a good opportunity where we come together and feedback to each other about what goals we’ve been working at, and then formulate a sort of more shared goal” (*Site 1)
		Electronic and paper-based record keeping systems	*“I spend more time doing paperwork than I actually do client work” *(Site 5)
			*“then every outcome measure is…put into a database and there’s a lot of crosschecking” (Site 5)*
	Beliefs about consequences	Lack of client involvement in case conference (.^b^)	*“it’s fragmented though, through EMR [electronic medical record]…plus each therapist will write up what their goals are, so there’s no actual framework where they’re all in one”* (Site 3)
			*“we probably don’t—in this service—have a platform where we all get together as an MDT with the patient and actually look at their goals and how they’re progressing”* (Site 3)
Shared decision-making	Knowledge Skills	Clinicians skills to facilitate goal-setting interactions (^a^/.^b^)	*“potentially for junior staff, they’re so overwhelmed… learning their discipline specific stuff, that to think broader can be quite hard” (Site 1)*
			*“I think, a big challenge for our team in particular, is that level of skill and experience with doing that very specific goal-setting and being able to break it down with the patient engaged in that process and show them how it all connects, and coming up with very specific goals that are still their goals” (Site 2)*
	Beliefs about consequences	Timing of goal-setting interactions (possibly too early inpatient rehabilitation) (.^b^)	*“We’re still grappling with, okay, this is what they’ve done in a few days since their stroke, we think this is what they might get to based on their type of stroke and experience …So if we’re grappling with it and we’re expecting them to have some kind of concept, it’s really hard to—to do it so early”* (Site 3)
	Beliefs about capabilities	Clinician challenges with those who have cognitive impairment or who are not ready to goal set (.^b^)	*“their cognition means that they are … insightless to their impairments and function so it ends up being therapist directed*” (Site 1)
	Environmental context and resources	Time taken to conduct goal-setting interactions (^b^) Service setting context (.^a^)	*“the amount of time it takes to do goal-setting well, which, here [community rehabilitation] … it’s a bit better because you can actually say, “Okay, I need to actually make an appointment time to do this. Whereas in inpatients, you’re lucky if you get two minutes to spend on goal-setting, realistically” (Site 4)*
		Using measurement tools and questionnaires (.^a^)	*“others who maybe are having a bit more trouble really getting out clear goals, or coming up with goals, then I might switch to a tool like the COPM or something, to help.”* (Site 4)
Meaningful and specific goals	Social influences	Clinicians obligation to set goals relevant to the role of the service (.^b^)	*“Or goals that are appropriate to our service, is harder to extract than just personal goals” (Site 2)*
			*“I think there can be some conflict between the patient’s own goals that are maybe set spontaneously without much guidance from therapists, and then from the service perspective” (Site 2)*
	Beliefs about consequences	Clinicians beliefs that goal-setting needed to be realistic to avoid client disappointment (.^b^)	*“cause otherwise you’re setting them up to fail” (Site 1)*
			*“it’s that fine line between them being something that the patient wants and something that we think is achievable”* (Site 1)
	Environmental context and resources	The differences in goal focus between inpatient and community rehabilitation settings (.^b^)	*“person centred goal-setting is this big board idealistic thing that sometimes we maybe struggle to achieve in the setting, because the setting dictates what you work on”* (Site 1)
Action planning and review	Knowledge	Understanding the differences between goals and actions or staff task lists (.^b^)	*“normalising their calcium, normalising their potassium, normalising whatever. Getting them pain-free. Getting on top of their pain is a goal” (Site 3)*
			*“we’ve got a whole pile of participation goals, activity goals, impairment goals and there’s some education goals or information, like information about EPOA [enduring power of attorney] and wills we pop … that can be a goal as well”* (Site 5)
	Memory attention and decision-making	Goal review and feedback was often ‘forgotten’ (.^b^)	*“people are generally quite good at going and getting the initial goals off people, but the review process of reviewing the goals and things, I don’t think happens consistently”* (Site 2)
	Environmental context and resources	Using charts to track progress and communicate goal actions (.^a^)	*“we will have their goals up on the wall and we will tick them off and its, it’s exciting for nurses and patients to see…. or well anyone who helps that patient… to see that it’s been ticked off, is a big achievement on everyone's behalf”* however staff identified this was a *“more reactive than proactive”* approach (Site 1)

(^a^) symbol indicates enabler, (.^b^) symbol indicates a barrier

#### Interdisciplinary teamworking

Clinicians’ knowledge and skill were identified as barriers to implementing interdisciplinary goal-setting (Knowledge and /or skill, TDF). Despite discussing the importance of client-centred goal-setting, rehabilitation clinicians were often unable to demonstrate how clients were involved in the goal-setting process beyond initial goal-negotiation. Furthermore, clinicians expressed concern about conducting goal-setting discussions with clients when the area of goal focus lay outside their specific discipline skillset.

Nursing staff were often not directly included in the goal-setting process but could identify the value that they would add “*because we carry out what everyone else plans for them*” (Site 1) (Social and professional role and identity, TDF). However, some nurses voiced that they had limited training in goal-setting (Skills, TDF) and it was not traditionally part of their role.

Having a structured process for case conferences, strong leadership and chairing enhanced interdisciplinary goal-setting (Environmental Context and resources, TDF). However, clients were not included in case conference discussions and no clinicians thought it was possible that the client could be involved in the case conference. A structured goal-setting process was identified as an enabler by clinicians. However, clinicians highlighted the need for these structures to be flexible to accommodate client needs, specifically in inpatient rehabilitation, when the length of stay between clients with different diagnosis varied considerably.

#### Shared decision-making

Shared decision-making during goal-setting requires a collaboration between the client and the healthcare professional to identify client values and preferences [36]. In focus groups, clinicians highlighted the importance of having the knowledge and skills to facilitate goal-setting discussions. Enablers of client-centred goal-setting interactions included motivational interviewing skills, active listening skills, skills to break longer term goals into shorter term goals and skills to support clients to create action plans to facilitate goal achievement (Knowledge and/or skill, TDF). Clinicians questioned the effectiveness of goal-setting early in inpatient rehabilitation expressing that both clinicians and clients were unprepared to set goals on admission to rehabilitation.

*“So, if we’re grappling with it and we’re expecting them to have some kind of concept, it’s really hard …to do it so early” (Site 3).*

*“So actually, is the goal-setting with them futile or not effective, because we’re doing it at the wrong time for that particular patient” (Site 1) (Beliefs about consequences, TDF).*

Clinicians found it difficult to facilitate goal-setting discussions when clients had cognitive and communication impairments or lacked motivation and confidence to participate. Whilst some clinicians demonstrated awareness that they may require additional training to facilitate goal-setting with clients who have cognitive and communication impairments (Skills, TDF), other clinicians believed it was not possible for these clients to set goals; “*by its very nature it is beyond the capacity of some of our clients”* (Site 5) (Beliefs about capabilities, TDF).

The time taken to facilitate shared decision-making goal-setting sessions was highlighted as a barrier. This was further hampered by lengthy assessments in one community rehabilitation service and constraints of both paper-based and electronic medical record keeping systems in all services (Environmental Context and resources, TDF). The inflexibility of electronic medical record keeping systems impacted the feasibility of setting common goals and actions as a rehabilitation team. Furthermore, including the client in team goal-setting discussions was considered too time consuming. Advantages of working in a community context meant that clinicians could segregate time specifically for goal-setting discussions.

Using measurement tools and structured questionnaires to prompt goal-setting discussions was an enabler that promoted client involvement (Environmental Context and resources, TDF). Clinicians highlighted that additional training may be required to use the tools, specifically when this was not common practice within their role; “*I actually find that MGAM [multidisciplinary goal attainment measure] really hard to do, to explain for people to understand how they can rate it”* (Site 5) (Skills, TDF)*.*

#### Meaningful and specific goals

Whilst all clinicians felt it was their role to set goals with clients in rehabilitation, they also felt obligated to set goals that aligned with the role of their service, and this was most obvious in inpatient rehabilitation (Social influences, TDF). Clinicians in one community rehabilitation service wanted to set hope or dream-based goals with clients but were concerned this could negatively reflect on the service, which used goal achievement as a service outcome measure (Beliefs about consequences, TDF). Clinicians believed that goal-setting could motivate patients and potentially impact client outcomes; “*[goal-setting] encourages motivation; patients are more motivated to participate in therapy, so we get better results…and potentially quicker throughput”* (Site 1). Conversely, other clinicians felt that goals set by rehabilitation clients needed to be realistic; *“cause otherwise you’re setting them up to fail”* (Site 1) (Beliefs about consequences, TDF).

The rehabilitation setting was perceived to have an impact on the type of goals that were set (Environmental Context and resources, TDF)*.* Community rehabilitation services highlighted how more meaningful goals could be established once discharged from an inpatient service when the client had been able to experience living back in a real-world environment. Whilst inpatient rehabilitation services highlighted a focus on getting clients out of hospital and the perception of organisational pressure to reduce lengths of stay.

*“it’s very clinician-driven in the inpatient setting…in the community setting, … they’re back at home, it’s more empowering, because they’re back in their normal environment”* (Site 4).

#### Action planning, feedback and review

There was limited knowledge and understanding demonstrated by clinicians on the difference between a goal and an action plan or task list (Knowledge, TDF). Clinicians lacked an understanding of the importance of client actions versus staff actions. Clinicians focussed on how they personally would assist goal achievement for the client with little consideration to empowering client’s goal pursuit through developing client action plans, appraisal of client actions and the delivery of feedback to the client. Two sites (Sites 2, 5) initiated a key worker role (prior to this research) as a primary point of contact, providing feedback for the client, albeit inconsistently, as clinicians reported they often forgot to feedback to the client. (Memory, attention and decision-making, TDF). Clinicians highlighted that review of goals was most often conducted to ‘measure’ goal-setting outcomes (predominantly for the benefit of the service), but clinicians didn’t highlight the value of reviewing client action plans or providing feedback about performance or results to clients.

Charts on walls to communicate patient goals were an enabler at one site (Site 1) (Environmental Context and resources, TDF). Charts were used to communicate the client’s goals to all members of the rehabilitation team, facilitate reinforcement of actions and demonstrate progress when goals were ‘ticked off’ throughout the admission. Whilst using charts on walls was reported as a valuable strategy by clinicians, the charts were not used consistently and relied on individual clinicians identifying that a client needed further encouragement and support from the team to participate in rehabilitation activities.

### Phase 2- adapt the knowledge to context, select, tailor and implement interventions—methods and results

Three co-design workshops were facilitated by the primary researcher (AB) at each site. The findings of phase 1 and a previous study [11] informed the co-design workshops supporting the adaptation, selection and tailoring of interventions. In keeping with a co-design approach both staff and consumer workshops were included in this phase of the study.

#### Participants

Medical staff, nursing and allied health professions were eligible to participate in the workshops if they were involved in goal-setting. Participants were able to be involved in both phases of this study, 28 clinicians across the 5 sites participated in both the focus groups and workshops. No other exclusion criteria were applied. A maximum number of 20 clinicians from each site participated across all co-design workshops in this study. A maximum of 20 clinicians were selected to ensure diverse representation of staff across professional groups, level of experience, inclusion of leadership roles within the rehabilitation team and to ensure workshops were easily facilitated. Written consent was provided by all clinicians. Demographic details of clinicians included in the co-design workshops are in Table 3.

**Table 3:** Demographics of co-design workshop participants

Site	Disciplines represented	Gender	Average Age	Average years of experience in the profession	Average years of experience in rehabilitation
Site 1 (*n* = 10) (1 missing data points)	Dietetics, Neuropsychology, Nursing, Physiotherapy, Rehabilitation Consultant, Speech Pathology	100% female	33.89 (± SD 7.66)	12 (± SD 8.63)	7.53 (± SD 6.05)
Site 2 (*n* = 12) (2 missing data points)	Neuropsychology, Nursing, Occupational Therapy, Physiotherapy, Rehabilitation Consultant, Social Work, Speech Pathology	100% female	39.80 (± SD 9.39)	14.25 (± SD 7.44)	9.00 (± SD 5.49)
Site 3 (*n* = 10) (1 missing data point)	Nursing, Occupational Therapy, Physiotherapy, Rehabilitation Consultant, Speech Pathology	20% male	40.00 (± SD 8.40)	15.56 (± SD 8.95)	9.44 (± SD4.90)
		80% female			
Site 4 (*n* = 14) (2 missing data points)	Allied Health Assistant, Dietetics, Neuropsychology, Nursing, Occupational Therapy, Physiotherapy, Speech Pathology, Social Work	100% female	39.82 (± SD 11.72)	12.64 (± SD 10.50)	7.82 (± SD 5.67)
Site 5 (*n* = 5) (1 missing data point)	Dietetics, Occupational Therapy, Speech Pathology	100% female	41.75 (± SD 14.24)	18.50 (± SD13.77)	16.70 (± SD 12.77)

Expressions of interest were sought from members of a local rehabilitation consumer group (already established at one of the sites) to participate in the consumer workshop. To allow all consumers to participate as desired a maximum of 10 consumers were identified to participate in the workshop. Informed signed consent was provided by all consumers.

#### Procedure

A maximum of two hours was allocated for each co-design workshop. The first two workshops involved iterative review of data from phase 1 and a previous study [11]. Data specific to each site were presented in PowerPoint presentations and infographics to promote discussion throughout the first co-design workshop. Ideas to bridge evidence-practice gaps using previously published goal-setting examples were discussed including; the goal-setting and action planning framework and client workbook [10], teamworking strategies [37, 38], shared decision-making aids [39], goal-setting education and training [40, 41] and audit and feedback [31]. As a component of the Behaviour Change Wheel questioning strategies used during the workshops to facilitate clinicians to a consensus agreement on interventions to be implemented were based on the APEASE criteria (Affordable, Practical, Effective/cost effective, Acceptable, Safe, Equitable) [30]. Following the TDF analysis of barriers and enablers in phase 1, the Behaviour Change Wheel (BCW) was then used to expand upon the findings to identify intervention functions and behaviour change techniques to be incorporated into the interventions developed and the implementation plans [27, 28]. All resources and tools were then designed by the primary researcher after the workshops and circulated back to each service via email for comment and further tailoring at the third workshop.

The third co-design workshop at each site aimed to develop an implementation plan that described; who will do what? How? When? And where? Process mapping activities conducted by the primary researcher during the co-design workshops assisted clinicians to identify individual tasks, roles and responsibilities for the implementation plan. Wherever able tasks in the implementation plan were incorporated into existing processes, prior to changing processes or adding additional processes. Data collected from each co-design workshop was in the form of researcher field notes.

One consumer group consultation session was run between staff co-design workshops 2 and 3 to facilitate feedback on consumer resources that clinicians had suggested. Not all consumers could attend the co-design workshop in person and further feedback was received via email.

A local site facilitator was funded (8 h a week) at each service to facilitate the implementation of interventions and was supported by the primary researcher. Local site facilitators planned to conduct regular audits of the interventions being implemented, provide prompts and feedback to the rehabilitation clinicians, provide education and revise interventions as required throughout implementation.

#### Results

The outcomes of phase 1 highlighted that to enhance the ‘active ingredients’ of goal-setting clinician’s knowledge, skills and beliefs about the consequences of goal-setting as well as the environmental context, social and professional roles of different team members needed to be addressed. The interventions co-designed to address these areas across the five sites included staff education and training modules, a key worker model, interdisciplinary goal-based case conference templates, communication goal boards, a client held workbook and educational rehabilitation service flyers. Table 4 outlines the content of co-designed interventions, the active goal-setting ingredient addressed, the TDF domain addressed within each intervention, the intervention functions identified, and the behaviour change techniques to be incorporated in each intervention [42]. The interventions were designed to allow each site to tailor relevant sections to their setting. Detail of the interventions developed are published online [42].

**Table 4:** Interventions co-designed to address TDF domains (Including intervention functions and behaviour change techniques

Interventions	Active goal-setting ingredient addressed	TDF domains addressed	Intervention Functions	Behaviour Change Technique
**Staff Education and Training sessions**	Interdisciplinary goal-setting Shared decision-making Specific and meaningful goal-setting Action Planning, feedback and review	Knowledge Beliefs about consequences Social Role and Identity Skills Beliefs about capabilities Social influences	Education Modelling Enablement Training	Information about health consequence Demonstration of the behaviour Instruction on how to perform the behaviour Behavioural practice/rehearsal Framing and reframing Self-monitoring of behaviour (audits) Feedback on behaviour (audits)
*Purpose:* To enhance understanding of;				
→ The difference between goals and actions, → How to frame long-term and short-term goals → Action plans and coping plans to achieve goals → Skills to facilitate SDM → How teams can work together to include the client → Using measurement tools and prompts				
*Modules:*				
→ An introduction to goal-setting → Collaborative interdisciplinary goal-setting → Goal-setting and Action Planning → Motivational Interviewing → Goal-setting measures and tools → Talking Mats training online *Materials:* Training manual, handouts, references and PowerPoint presentations*,* online resources				
**Keyworker model and workplace instruction**	Interdisciplinary goal-setting Shared decision-making Specific and meaningful goal-setting Action Planning, feedback and review	Skills Environmental Context Social Role and Identity	Environmental Restructure Training	Restructuring the social environment Instruction on how to perform the behaviour
*Purpose:* To enhance client and family involvement in goal-setting and rehabilitation				
*Content:* Workplace instruction providing description of role and responsibilities				
*Materials:* Workplace instruction, orientation training				
**Case conference restructure**	Interdisciplinary goal-setting Specific and meaningful goal-setting Action Planning, feedback and review	Environmental Context	Environmental Restructure Enablement	Restructuring the social environment (processes) Framing and reframing Prompts and cues
*Purpose:* To provide a framework for meaningful interdisciplinary goal-setting and action planning				
*Content:* Background client information, current function, common goal focus, long term hope/dream goals, goals for the episode of care, weekly goals, client actions, staff actions and discharge planning				
*Materials:* Electronic template to be pasted into medical records, chairing guidelines, workplace instructions defining roles and responsibilities of team members				
**Communication goal and action boards**	Interdisciplinary goal-setting Shared decision-making Action Planning, feedback and review	Environmental Context	Environmental Restructure	Prompts and cues Restructuring the physical environment
*Purpose:* To enhance client and family involvement in goal-setting and rehabilitation, to enhance the role of the nurse to support action planning				
*Content:* Long term goals, goals for the episode of care, weekly/short term goals, client actions and estimated discharge dates				
*Materials:* Paper copy				
**Client workbook**	Shared decision-making Specific and meaningful goal-setting Action Planning, feedback and review	Knowledge Skills	Education Training	Information about health consequence Instruction on how to perform the behaviour
*Purpose:* To enhance client knowledge and expectations, to provide a framework for staff to facilitate goal-setting				
*Content:*				
→ What is rehabilitation? → Why do I need rehabilitation? (fillable) → Who may I meet in rehabilitation? → Your rehabilitation service (tailorable) → What is a goal? → How do I set a goal? (tailorable templates)				
*Materials:* Editable PDF, provided as hard copy to clients				
**Client Rehabilitation flyer**	Shared decision-making Specific and meaningful goal-setting	Knowledge	Education	Information about health consequence
*Purpose:* To enhance client knowledge and expectations *Content:*				
→ What is rehabilitation? → What is a goal? → Who may I meet in rehabilitation? (tailorable) → Available facilities (tailorable) → What to bring (tailorable) → Your rehabilitation routine (tailorable)				
*Materials:* Editable PDF, provided as hard copy to clients				

#### Interventions

All interventions were considered safe and practical by all sites. All sites felt that selected interventions should be delivered equitably to all clients in the service, despite clinician’s concerns about clients differing needs. Each site chose to implement one or more of the interventions based on what was feasible in their current context and what they felt would most improve their goal-setting practices. All sites chose education and training as an intervention to enhance clinicians understanding of the active goal-setting ingredients. Clinicians at Site 1 chose to implement changes to case conference and to utilise formal client goal boards. Site 2 chose to implement all interventions and embed many of these within their existing key worker role. Clinicians in Sites 3,4 and 5 chose to implement the client held workbook and designed specific templates for team goal-setting and action planning. Site specific implementation plans were developed to support uptake of selected resources and strategies (Table 5).

**Table 5:** Site specific Implementation Plans

Site	Interventions	Who	What	How	When	Where
Site 1	Case conference restructure & guidelines	MDT meeting (Client not present)	Formulation of common goals, staff actions and client actions	Face to Face group	First case conference (Client not present) Weekly thereafter	Meeting room on ward
	Client goal boards	Nominated team member and client	Discuss with the client and display goal boards in room	Prepopulated from case conference	After each case conference	Rehabilitation ward
	Staff education and training	Site facilitator	Goal-setting education, workplace process education	PowerPoint presentations, case discussions and 1:1 sessions	Prior to and throughout implementation/on orientation to service	Meeting room
		Two nominated staff	Talking Mats Training [41]	Online training and assignments	During implementation	Online
Site 2	Client rehabilitation flyer	Intake nurse	Educational brochure	Face to face	Prior to admission	Acute hospital wards
	Goal-setting workbook	Administration officer	Print client booklets	On ward computers	Prior to admission	Rehabilitation reception
		Admitting nurse	Give client booklet	Face to face	On admission to rehabilitation ward	At client bedside
		Link worker (Key worker) and client	Goal-setting and rehabilitation education and goal negotiation	Face to face	Within 48 h of admission	Rehabilitation ward
	Case conference restructure & guidelines	MDT (Client not present)	Formulation of common goals, staff actions and client actions	Chaired by consultant/NUM or Advanced AHP	First case conference and weekly thereafter	Meeting room on ward
	Client goal boards	Key worker and client	Discuss with client and display goals and actions on goal board in room	Prepopulated from case conference documentation	After each case conference	Rehabilitation ward
	Staff education and training	Delivered by the site facilitator	Goal-setting education, process education and workplace instruction	PowerPoint presentations, case discussions and 1:1 sessions	Prior to and throughout implementation/on orientation to service	Meeting room on ward
		Two nominated staff	Talking Mats Training	Online training and assignments	During implementation	Online
Site 3	Goal-setting workbook	Admitting nurse	Long term goal-setting discussions, and goals for the episode of care set	Documented using the goal-setting workbook	On admission to rehabilitation ward	Rehabilitation ward
		All disciplines	Complete -How do I set a goal? Templates and action plans	Documented using the goal-setting workbook	On initial assessments and updated in treatment sessions thereafter	Rehabilitation ward
	Staff education and training	Delivered by the site facilitator	Goal-setting and rehabilitation education and goal negotiation skills	PowerPoint presentations, case discussions and 1:1 session	Prior to and throughout implementation/on orientation to service	Meeting room
		Two nominated staff	Talking Mats Training	Online training and assignment	During implementation	Online
Site 4	Goal-setting workbook	Intake nurse	Goal-setting discussions, long term goal-setting and goals for the episode of care discussed	Documented using the goal-setting workbook	On intake assessment	Consultation room
		All disciplines	Weekly goals and actions set, reviewed and updated	Documented using the goal-setting workbook	On initial assessments and follow up reviews	Consultation rooms or therapy areas
	Staff education and training	Delivered by the site facilitator	Goal-setting and rehabilitation education and goal negotiation skills	PowerPoint presentations, case discussions and 1:1 sessions	Prior to and throughout implementation/on orientation to service	Meeting room
		Two nominated staff	Talking Mats Training	Online training and assignments	During implementation	Online
Site 5	Goal-setting workbook	Care Coordinator (Key worker)	Goal-setting discussions, and goals for the episode of care discussed	Documented using the goal-setting workbook	On initial assessment	Home visit or consultation room
		All disciplines	Weekly goals and actions set	Documented using the goal-setting workbook	On initial assessments and follow up reviews	Home visit or consultation room
	Staff education and training	Delivered by the site facilitator	Goal-setting and rehabilitation education and goal negotiation skills	PowerPoint presentations, case discussions and 1:1 sessions	Prior to and throughout implementation/on orientation to service	Meeting rooms
		Two nominated staff	Talking Mats Training	Online training and assignments	During implementation	Online

#### Education and training

It was unanimously agreed that clinicians would benefit from education and training to address all of the ‘active ingredients’ of goal-setting. Five education modules were developed for sites to select from based on what was relevant for their setting and skill mix (Table 4). Training in the use of Talking Mats [43] was offered to two clinicians at each site. Talking Mats are a low-tech communication tool that has been developed to assist clinicians in communicating with clients with aphasia or cognitive impairment. Training in the use of this resource was implemented to enhance clinician’s skill in facilitating goal-setting discussions for clients with communication difficulties. Education and training would be delivered through a combination of online recorded orientation modules, face to face sessions delivered by a local site facilitator during implementation and one on one sessions as required.

#### Keyworker model

Site 5 continued to operate a keyworker role conducting goal-setting on admission to the service, reviewing goals throughout the admission and completing measurement of goal achievement on discharge. Site 2 chose to build on an existing keyworker role by including feedback and review of goals with clients prior to and following case conference. Site 4 also chose to expand their intake role to incorporate goal feedback pre- and post-case conference. In both services these roles had previously been responsible for supporting goal-setting on admission to the service.

#### Case conference restructure

Modifying the case conference formatting was not universally accepted, as this forum was already a lengthy process and changes to goal-setting practices in this forum were considered too time consuming by several sites. Case conferences were conducted at all sites and are used as a forum for discussing client’s current function, progress and discharge planning. Two sites (Site 1 and 2) adopted changes to case conference. Both sites felt that the case conference forum, presented the best opportunity to develop a common goal focus. However, neither site chose to involve the client in case conference due to concerns about the time involved and challenges arising when having difficult conversations in front of clients. In response, Site 1 nominated clinicians to provide feedback to clients following the case conference and ensure goals reflected individual client preferences, whilst Site 2 chose to utilise the keyworker role as a client advocate in case conference and to translate information discussed at case conference with the client.

#### Client goal boards

Two sites (Sites 1 and 2) chose to implement client goal boards to communicate goals and actions set in case conference with the client, family and wider rehabilitation team. To avoid duplication of documentation, the goal boards were designed to pre-populate from case conference documentation and clinicians wrote the goals in client appropriate language on A4 or A3 paper. The goal boards were placed on the client’s wall (as consented by the client) following case conference and Site 2 planned to place an additional copy into the client workbook.

#### Client workbook

Four sites (Site 2,3,4,5) chose to implement a client workbook to; provide education to clients about goal-setting, provide a framework to support clinicians during goal-setting, and to document goals and actions set. However, some clinicians expressed that previous experiences trying to implement a client workbook had been unsuccessful. One community rehabilitation service suggested development of a mobile phone application to facilitate client goal-setting and action planning across the team however this was beyond the resources of this study. Site specific processes to implement the client workbook were developed based on clinicians’ roles and responsibilities (see Table 5). Each site requested slightly different goal-setting and action planning templates and therefore six templates were designed that could be further tailored and inserted into the goal-setting workbook. Plans were made at each site to define who would print the workbooks, where they would be kept, who would give them out to clients and who was responsible for completing specific sections (Table 5).

#### Client rehabilitation flyer

Clinicians at Site 2 felt that clients needed more preparation for goal-setting prior to their arrival on the inpatient rehabilitation ward. However, there were concerns that the client workbook would be lost on the acute wards or in transition to rehabilitation if it was given prior to admission. Therefore, a rehabilitation flyer was developed to supplement the workbook information in a more cost-effective manner. The flyer was to be given to clients accepted into the inpatient service during their assessment for rehabilitation.

## Discussion

Barriers and enables to implementing active goal-setting ingredients aligned with a range of TDF domains. Firstly, and most prominently, the environmental context and resources of workplaces was identified as both a barrier and enabler. Clinicians also demonstrated a lack of fundamental knowledge about the different components of rehabilitation goal-setting, specifically the difference between goals and actions. This was complicated by tensions between clinicians perceived obligations to set goals that aligned with organizational priorities rather than the priorities of the client. Other keys barriers and enablers related to clinician skills to implement shared decision-making and some clinician’s beliefs about the consequences associated with goal-setting. The interventions developed and the site-specific implementation plans in this study were influenced by these identified TDF domains, which were mapped against the BCW intervention functions and behavior change techniques.

All sites chose to include staff training and education sessions into their implementation package. Education and training sessions were targeted to enhance clinicians’ knowledge, specifically focusing on understanding the components of goal-setting including action planning, feedback and review and ensuring that clinicians had the skills to set specific and meaningful, client centred goals. Clinicians in this study frequently misunderstood the difference between a goal and an action, often identifying staff actions as rehabilitation goals. The confusion of actions and goals may reflect a clinicians focus on health system expectations rather than client’s goals [19]. When clinicians discussed goals, these were often framed to reflect the needs of the organisation rather than the client; i.e. ‘getting them out of hospital’ rather than ‘the client going home to live with their family’.

In keeping with previous research [19], clinicians in this study also spoke of the pressure to be able to accurately predict recovery to set *realistic* goals with clients. Conversely, consumer voiced literature emphasises the need for clinicians to include hope and dream based goals in rehabilitation to enhance the motivational aspects of goal-setting [44]. Whilst some clinicians acknowledged that goal-setting can be motivational they still felt that goals should be realistic. If clinicians continue to only set ‘realistic goals’ with clients and not include aspirational goals, it is likely that goals will remain subjective to the clinician’s expectation for recovery and therefore unlikely to enhance the motivational aspects of goal-setting that may impact client outcomes.

The focus on setting goals to meet perceived organisational needs and the inflexibility in clinicians’ perspectives of including aspirational goal-setting led to clinicians demonstrating a therapist led model of goal-setting. The therapist led model of goal-setting has been seen previously in Queensland rehabilitation services [45]. To move forward from a therapist-led model of goal-setting, education and training should focus on supporting clinicians to engage clients in shared decision-making to set client-centred goals and to clinically reason to support inclusion of tangible short-term goals and longer term aspirational goals across the rehabilitation journey. In this study, the client workbook and case conference templates were designed to facilitate setting of longer-term aspirational goals and allow shorter term goals to be broken down from these longer-term goals.

The client workbook was designed to ensure a common client centred goal focus was established among the team (including long term and short-term goals) and that components of action planning and review were included in the goal-setting process. The client workbook was designed to provide information to clients to enhance their knowledge and to assist clinicians to develop skills to structure their goal-setting discussions with clients. Therefore, the client workbook was developed to target both client and clinician behaviours. Developing interventions to target both the client and clinician is a novel approach in rehabilitation research with previous studies suggesting that it may enhance shared decision-making more than targeting only one group [36].

Of interest in this study was the way in which rehabilitation teams chose to work together and include the client to achieve an interdisciplinary common goal focus. The case conference format was redesigned to include a common goal focus rather than discipline specific reporting and to include specific staff and client actions. In this study two sites chose to use the case conference forum (including feedback to the client) to achieve a common, team goal focus. Interdisciplinary models of rehabilitation involve clients as a member of the team however, clinicians in this study were unwilling to involve the client with the team in goal-setting discussions within the case conference. There is limited literature about the inclusion of clients in rehabilitation case conference meetings. One study reported that goal-setting ward rounds that include the client were more beneficial than meetings without the client [46]. However, previous studies have found barriers to including clients in team meetings including; a lack of clinician skill and confidence to facilitate team discussions in goal-setting meetings with the client present [21] and a belief that this approach would be too time consuming [47]. When reviewing the time commitment involved in including the client in goal-setting meetings one study showed that clinicians spent over 4 h longer each week in goal-setting meetings that included the client than those without the client [47]. To ensure goal-setting is truly interdisciplinary the client should be considered a member of the rehabilitation team and be included in shared decision-making at each point in the goal-setting process. How this can be achieved in a cost-effective manner with or without the client attending team meetings requires further research. One strategy suggested in this study was to introduce a further expansion on the key worker role to ensure dedicated time was set aside to relay goal-setting information discussed in the case conference with the client.

Three sites implemented or built on existing key worker roles to enhance communication and include feedback channels to clients. A lack of time was reported as the biggest barrier to implementing the key worker role. Clinicians across all sites were concerned that adding specific these roles to their clinical practice may detrimentally impact their workloads. In this study clinician’s suggested strategies such as pre-population of the client goal boards to reduce time spent repeating documentation, whilst case conferences were monitored to evaluate time taken to introduce the changes. In Site 2 the client workbook was given on admission to give clients time to read the resource and ensure key worker discussions could be facilitated quicker. Concerns about a lack of time to conduct goal-setting activities has also been reported in many other rehabilitation goal-setting studies [8, 21, 25, 48]. Clinicians in this study demonstrated a perception that organisational priorities and length of stay presented a barrier to having more time for engaging clients in goal-setting discussions. Some clinicians referenced their lack of staff resourcing as a reason for being time poor in rehabilitation settings. A previous study undertaken in the United Kingdom found staffing ratios as well as time spent in information exchange and in non-clinical activities significantly impacted the face-to-face therapy delivered to stroke survivors [49]. Whilst many Queensland rehabilitation services have staffing levels below recommended national benchmarks [50, 51], how Queensland rehabilitation services are structured and how clinicians structure their day-to-day clinical practice to maximise time for goal-setting discussions is unclear.

Supporting the need for a co-design approach clinicians at all sites stated that they had different requirements for goal-setting interventions and needed to tailor interventions to their service delivery models and different service settings. Also of interest is the number of interventions sites chose to implement with some sites choosing one intervention in addition to education and training and another site choosing to implement all interventions to complement each other. There is little evidence to support implementation of multifaceted interventions over single interventions.

The co-design process was hypothesised to engage staff and to enhance ownership over goal-setting interventions. Sites within this study demonstrated differing levels of engagement and required different levels of direction to design and tailor goal-setting interventions. All teams in this research study required substantial facilitation throughout the co-design workshops to define solutions to intervention challenges, to develop implementation plans and allocate roles and responsibilities to individual team members. Healthcare services should consider the need for funding, as well as the level of facilitation skills required by individuals to support change when aiming to develop holistic, theoretically informed co-designed healthcare interventions. Further research to explore the impact of these interventions in clinical practice and how teams go about implementing the interventions is warranted.

## Conclusion

Strengths of this study include the use of a holistic conceptual framework for goal-setting in rehabilitation [11] and the use of co-design alongside an implementation process model (KTA) and determinant frameworks (TDF and BCW) to support intervention development and provide a guide for implementation plans [52]. Overall, the barriers and enablers to implementing the active ingredients of goal-setting in Queensland rehabilitation services are consistent with internationally published research. The framework presented for developing the specific interventions and tools throughout this study can support other teams to evaluate their goal-setting practices and enhance the active ingredients of goal-setting that have the potential to impact client outcomes and autonomy in rehabilitation.

## Supplementary Information

**Additional file 1.**

**Additional file 2.**

## Data Availability

The datasets used and/or analysed during the current study are available from the corresponding author on reasonable request.

## Abbreviations

APEASE: Affordability, Practicability, Effectiveness, Acceptability, Side-effects and safety, Equity
BCT: Behaviour Change Technique
BCW: Behaviour Change Wheel
KTA: Knowledge to Action Framework
MGAM: Multidisciplinary Goal Attainment Measure
MRC: Medical Research Council
SDM: Shared decision-making
TIDIER: Template for the Intervention Description and Replication Checklist
TDF: Theoretical Domains Framework
UK: United Kingdom
