# Supplementary material for: Developing tailored theoretically informed goal-setting interventions for rehabilitation services: a co-design approach
Source: BMC Health Serv Res. 2022 Jun 22;22:811. doi: 10.1186/s12913-022-08047-6 (PMC9214993; doi:10.1186/s12913-022-08047-6)
Supplement: Supplementary file 2 — Additional file 2. [file 12913_2022_8047_MOESM2_ESM.doc]

| Statewide Rehabilitation Clinical Network Patient-Centered Interdisciplinary Goal Setting Project |  |
| --- | --- |

# Focus Group Interview Guide

Can you tell me what person centered goal setting means to you and your rehabilitation service?

Can you tell me about the goal setting process that currently occurs here in your rehabilitation service?

Can you tell me the pros/cons of the goal setting process within your service?

- Prompt- Can you give me an example of a time when the goal setting process worked well?
- Prompt – Can you give me an example of a time when the goal setting process did not work so well?

If you were undertaking a service improvement, what would you retain within your goal setting process? What would you improve in your goal setting process?
